# Morphological Effects in Visual Word Recognition: Children, Adolescents, and Adults

**DOI:** 10.1037/xlm0000485

**Published:** 2017-09-28

**Authors:** Nicola Dawson, Kathleen Rastle, Jessie Ricketts

**Affiliations:** 1 Department of Psychology, Royal Holloway, University of London

**Keywords:** morphological decomposition, lexical decision, visual word recognition, adolescents, cross-sectional

## Abstract

The process by which morphologically complex words are recognized and stored is a matter of ongoing debate. A large body of evidence indicates that complex words are automatically decomposed during visual word recognition in adult readers. Research with developing readers is limited and findings are mixed. This study aimed to investigate morphological decomposition in visual word recognition using cross-sectional data. Participants (33 adults, 36 older adolescents [16 to 17 years], 37 younger adolescents [12 to 13 years], and 50 children [7 to 9 years]) completed a timed lexical-decision task comprising 120 items (60 nonwords and 60 real word fillers). Half the nonwords contained a real stem combined with a real suffix (pseudomorphemic nonwords, e.g., *earist*); the other half used the same stems combined with a nonmorphological ending (control nonwords, e.g., *earilt*). All age groups were less accurate in rejecting pseudomorphemic nonwords than control nonwords. Adults and older adolescents were also slower to reject pseudomorphemic nonwords compared with control nonwords, but this effect did not emerge for the younger age groups. These findings demonstrate that, like adults, children and adolescents are sensitive to morphological structure in online visual word processing, but that some important changes occur over the course of adolescence.

The ability to recognize words rapidly and automatically is fundamental for skilled reading. Research on reading acquisition has focused primarily on the influence of phonological processing (Melby-Lervåg, Lyster, & Hulme, 2012), but there is also evidence that semantics (see Taylor, Duff, Woollams, Monaghan, & Ricketts, 2015 for a review) and morphology (Carlisle & Stone, 2005; Mahony, Singson, & Mann, 2000) have an important role to play. In children, the contribution of morphological knowledge to reading increases beyond the 4th grade (Singson, Mahony, & Mann, 2000), and by adulthood, the recognition of printed words involves rapid decomposition of morphologically structured words (Rastle et al., 2004). Yet despite wide evidence of explicit morphological awareness in children as young as seven years (e.g., Kirby et al., 2012), it is not known when this knowledge becomes implicit and automatized. This article reports the first study to track online morphological processing from childhood, through adolescence and into adulthood.

Morphological knowledge does not develop uniformly. Evidence suggests that derivational morphology develops over a more protracted period relative to inflectional morphology (Anglin, Miller, & Wakefield, 1993), and that explicit derivational knowledge continues to develop beyond 7th Grade (Nagy, Diakidoy, & Anderson, 1993). Despite this, comparatively few studies have investigated the influence of morphological knowledge on word recognition beyond Grade 5. English spellings depend on morphemic as well as phonemic units, so knowledge of morphology can help to resolve some of the apparent irregularities in the mappings between phonology and orthography and contribute to efficient recognition of complex words (Nagy, Berninger, & Abbott, 2006). This may be particularly important once knowledge of grapheme-phoneme correspondences is consolidated, as these connections can be chunked into larger units such as morphemes (Ehri, 2005). As children move through the education system, the types of words they encounter are increasingly comprised of multiple, and often layered, morphemic units (Nagy & Anderson, 1984; Nagy, Townsend, Lesaux, & Schmitt, 2012). Therefore, recognition of morphologically complex words becomes progressively more important for learning through reading and access to the curriculum.

One way to approach the development of morphological knowledge is to distinguish between implicit (or tacit) and explicit morphological processes (Goodwin, Petscher, Carlisle, & Mitchell, 2015; Nagy, Carlisle, & Goodwin, 2014). Explicit morphological knowledge is generally measured through tasks that tap morphological awareness, in which readers consciously analyze and manipulate morphemes in words (Carlisle, 1995). Tacit morphological knowledge is acquired implicitly through language learning and repeated exposure to morphemes across different contexts (Goodwin et al., 2015). According to Nagy et al. (2014), tacit morphological knowledge may contribute to word recognition both by creating stronger links between orthography, phonology and semantics, thus improving quality of lexical representations, and through the process of ‘chunking’ (see also Ehri, 2005), in which morphemes are processed as familiar units during recognition. Therefore, this aspect of morphological knowledge may be central to the development of the rapid, automatic word recognition processes characteristic of skilled readers.

The nature of morphological processing in visual word recognition has been much debated (Amenta & Crepaldi, 2012). Specifically, there is dispute over the processes by which morphemes are recognized in words. Proponents of morpho-orthographic theories argue that complex words are automatically decomposed on the basis of apparent morphological structure prior to lexical access (Rastle & Davis, 2008; Taft, 2004); others hold the view that morphological structure is analyzed once whole-word lexical access has occurred (e.g., Giraudo & Grainger, 2001). A third approach posits a parallel dual-route process, in which both whole-word access and decomposition are available (e.g., Baayen, Dijkstra, & Schreuder, 1997). The way that morphologically structured nonwords (e.g., *earist*) are processed poses an interesting question for these theories. By definition, nonwords are not represented in the lexicon, so evidence of decomposition in the recognition of these items is difficult to account for on the basis of postlexical morphological analysis (e.g., McCormick, Brysbaert, & Rastle, 2009).

A large number of studies have revealed morphological effects when adults process words and nonwords. Taft and Forster (1975) showed that nonwords comprising combinations of existing prefixes and stems (e.g., *dejuvenate*) were more difficult to reject than nonwords with existing prefixes and novel stems (e.g., *depertoire*), evidenced by increased response latencies and errors. This ‘morpheme interference effect’ was taken as evidence that morphological decomposition occurs prior to lexical access, as longer response latencies for *dejuvenate* nonwords reflect the additional process of checking the legitimacy of the prefix-stem combination once the stem has been isolated and identified. For novel stems (*pertoire*), this step is unnecessary as no lexical entry is found. More recently, support for the idea of prelexical morphological decomposition has come from eye tracking (Andrews, Miller, & Rayner, 2004), event related potential (ERP; Lavric, Clapp, & Rastle, 2007) and masked priming (Beyersmann, Castles, & Coltheart, 2011; Crepaldi et al., 2010; Rastle et al., 2004) studies.

Despite the wealth of evidence that skilled adult readers automatically decompose morphologically structured words and nonwords, few studies have addressed online visual processing of complex words in developing readers. This is important to inform theories of visual word processing in relation to morphology, and establish the developmental trajectory of automatized morphological knowledge. Studies have shown that children from around seven years of age demonstrate both tacit (e.g., Carlisle & Stone, 2005) and explicit (e.g., Kirby et al., 2012) morphological knowledge. For example, Carlisle and Stone (2005) investigated the impact of morphological structure on the speed and accuracy of word reading in 39 children aged 7 to 9 years (Grades 2 and 3) and 33 children aged 10 to 12 years (Grades 5 and 6). They compared responses to disyllabic derived words (e.g., *hilly*) with responses to monomorphemic ‘pseudoderived’ words matched on number of syllables, spelling and word frequency (e.g., *silly*). Both age groups were more accurate reading aloud the derived words compared to the pseudoderived words, providing evidence that morphological structure facilitates word reading in readers as young as seven years. Other studies have revealed similar findings (Burani, Marcolini, De Luca, & Zoccolotti, 2008; Laxon, Rickard, & Coltheart, 1992), but word naming as a measure depends on verbal output and is potentially subject to confounding factors such as articulation skill. Online measures such as lexical decision tasks better capture the automatic processes underlying visual word recognition.

Some researchers have used online paradigms to investigate morphological decomposition in developing readers, but findings have been mixed (Beyersmann et al., 2012; Burani et al., 2002; Casalis, Dusautoir, Colé, & Ducrot, 2009; Casalis, Quémart, & Duncan, 2015). Evidence from masked priming suggests that English children aged 7 to 10 years do not “blindly” decompose words that appear to have a morphological structure as adults do (Beyersmann et al., 2012), but studies with French (Quémart, Casalis, & Colé, 2011) and Hebrew-speaking (Schiff, Raveh, & Fighel, 2012) children have provided evidence for morpho-orthographic decomposition in young readers. Several studies have observed differences in how children respond to nonword stimuli with versus without morphological structure. For example, Burani et al. (2002) used a lexical-decision task with Italian children aged 8, 9 and 10 years and a group of adult controls, and found that accuracy was lower for morphologically structured nonwords compared to nonmorphologically structured nonwords in all groups, providing some evidence of a morpheme interference effect in children. Importantly though, stimuli across the two nonword conditions were poorly matched, with embedded stems present only in the morphological condition (e.g., *mammista*, the equivalent of *motherist* in the morphological condition was matched with *memmosto*, containing a nonword stem, in the nonmorphological condition). It is therefore unclear whether lower accuracy in the morphological condition was due to interference from the suffix, in line with previous findings (e.g., Crepaldi et al., 2010), or due to recognition of an existing stem. In the present study, stimuli were closely matched by adopting morphological and nonmorphological nonwords that share an existing stem.

The influence of morphological structure on children’s processing of words and nonwords has been demonstrated using online tasks in several languages such as French (Quémart, Casalis, & Duncan, 2012), Spanish (Lázaro, Camacho, & Burani, 2013), Dutch (Perdijk, Schreuder, Baayen, & Verhoeven, 2012) and Italian (Burani et al., 2002), but there is variation in how this effect emerges. For example, in lexical decision tasks involving real words, the presence of a stem slows word recognition in English but not French children, leading to the suggestion that English children are sensitive to embedded words while French children respond to the combination of morphological units (Casalis et al., 2015). In Spanish, complex words containing high frequency bases were recognized more quickly than those with low frequency bases, but this effect did not emerge in accuracy and was only seen in the most skilled readers (Lázaro et al., 2013). On the contrary, Perdijk et al. (2012) only found facilitatory effects of morphological family size on word recognition in less skilled readers.

In one cross-linguistic study on morphological effects in word recognition, Casalis et al. (2015) investigated word and nonword recognition in English and French children aged 7 to 10 years. Using a lexical-decision task, they showed that while the presence of morphemes supported recognition of words and impeded the ability to reject nonwords in all children, this emerged across accuracy and response latencies for French children, but only in accuracy for English children. While Casalis et al. (2015) report that their real word stimuli were matched for frequency, length and suffixes across languages, they do not state whether they accounted for variation in orthographic familiarity between the nonwords with and without suffixes. This leaves open the possibility that the morphologically structured nonwords were simply more ‘wordlike’ due to other factors, such as greater orthographic neighborhood size (Perea, 2000). Furthermore, across both nonword types there was inconsistency in orthographic transparency. For example, the nonword *namy* combined the root *name* with the suffix *y* (orthographic shift), yet other items (e.g., *waitery*) preserved the orthography of the root. While this is representative of the way derivational morphemes attach to stems in both English and French, there is evidence that children process words with an orthographic shift differently to words in which the stem is preserved (Lázaro, García, & Burani, 2015), yet this was not controlled across languages or stimuli. The present study addresses these issues by matching morphologically- and nonmorphologically structured nonwords pairwise on length, summed log bigram frequency and number of orthographic neighbors, and ensuring orthographic transparency across all items.

In summary, there is substantial evidence that complex words and nonwords are rapidly and automatically processed on the basis of morphological structure by skilled adult readers. At what stage in reading development this level of automaticity is reached is unknown. Children from around the age of seven demonstrate explicit morphological knowledge (Kirby et al., 2012), and there is growing evidence that they are also implicitly sensitive to morphological structure (Burani et al., 2002; Casalis et al., 2015). However, there appear to be qualitative differences in the way children process complex words compared with adults (Beyersmann et al., 2012). Conclusions from developmental research are further complicated by the variety of languages in which these studies have been conducted. Cross-linguistic generalizations are problematic because morphological structure may be processed differently in English compared to languages with less complex mappings between spelling and sound (Italian) or a richer system of derivational morphology (French).

One conspicuous omission in the current literature are online data from adolescent readers. This is important if we are to address the differences in morphological processing between children and adults, and track the emergence of adult-like morphological processing in visual word recognition. The present study investigates morphological decomposition in children (7 to 9 years), younger adolescents (12 to 13 years), older adolescents (16 to 17 years) and adults, using a visual lexical-decision task to probe processing of morphological and nonmorphological nonwords. Our cross-sectional design allowed us to examine developmental changes as individuals become skilled word readers. Including two adolescent groups allowed us to take a relatively fine-grained approach to investigating morphological effects during a time when much of the complexity in words that are encountered is driven by morphological structure (Nagy & Anderson, 1984) and knowledge of derivational morphology continues to grow (Carlisle, 1988).

Following Crepaldi et al. (2010), we hypothesized that adults would make more errors and show longer reaction times (RTs) when rejecting nonwords comprising a stem and suffix (pseudomorphemic nonwords) relative to nonwords comprising a stem and nonmorphological ending (control nonwords). We predicted that if children are also sensitive to morphological structure, then they too would show lower accuracy for pseudomorphemic nonwords compared to control nonwords. It was less clear whether this effect would emerge in their RTs, as previous findings have been mixed (Burani et al., 2002; Casalis et al., 2015). While there is no existing evidence that adolescents show a morpheme interference effect in their responses to morphologically structured nonwords, previous studies have indicated sensitivity to morphological structure in this age group (Goodwin, Gilbert, & Cho, 2013), so we expected to see processing costs in response to pseudomorphemic nonwords.

## Method

### Participants

Participants comprised 50 children (7 to 9 years, *M* age = 8.39, *SD* = .58; corresponding to 3 to 5 years of formal literacy instruction; 20 female) and 37 younger adolescents (12 to 13 years, *M* age = 12.67, *SD* = .31; corresponding to 8 to 9 years of formal literacy instruction; 18 female) recruited from mainstream primary and secondary schools, 36 older adolescents (16 to 17 years, *M* age = 17.04, *SD* = .32; corresponding to 12 to 13 years of formal literacy instruction; 24 females) recruited from schools and at a school event run at Royal Holloway, University of London, and 31 adults (*M* age = 20.12, *SD* = 1.56; 24 female) who were undergraduate and postgraduate students attending Royal Holloway, University of London. None of the participants had a recognized special educational need, and all spoke English as their first language. Adult participants were paid £5 for their time and travel expenses. The study was approved by the Psychology Departmental Ethics Committee at Royal Holloway, University of London.

### Materials and Procedure

Background measures were conducted to characterize the sample. Participants completed standardized assessments according to manual instructions in one session, and prior to the experimental task.

#### Nonverbal ability

This was measured using the Matrix Reasoning subtest of the Wechsler Abbreviated Scale of Intelligence–Second Edition (WASI-II; Wechsler, 2013), which is a pattern completion task.

#### Oral vocabulary

This was measured using the Vocabulary subtest of the WASI-II (Wechsler, 2013) for which participants are asked to verbally define words.

#### Word reading

This was assessed using the Sight Word Efficiency (SWE) and Phonemic Decoding Efficiency (PDE) subtests of the Test of Word Reading Efficiency–Second Edition (TOWRE-2; Torgesen, Wagner, & Rashotte, 2012) for which participants read aloud a list of words (SWE) or nonwords (PDE) as quickly as they can in 45 seconds.

#### Lexical-decision task

##### Stimuli

The stimuli comprised two sets of nonwords (30 pseudomorphemic and 30 control, see the Appendix) and two sets of words (30 morphologically complex and 30 monomorphemic), giving a total set of 120 items (drawn from Crepaldi et al., 2010). The words were used as filler items to balance the number of words and nonwords in the task. They were not further analyzed because: a) previous findings regarding the influence of morphological structure on real word recognition in lexical decision tasks have been mixed (Casalis et al., 2015; Quémart et al., 2012); b) it would be necessary to account for the changing influence of psycholinguistic factors (such as frequency and number of orthographic neighbors) across age; c) the words were not as closely matched across condition as the nonwords (e.g., the stems of the complex words did not overlap orthographically with the monomorphemic items). In the pseudomorphemic condition, English stems were paired with English suffixes (e.g., *earist*) to create a syntactically legal nonword. The control nonwords were created by pairing the same stems with a nonmorphological ending (e.g., *earilt*). These endings were formed by changing one letter of the morphological suffixes used in the pseudomorphemic condition; thus, there was a high level of orthographic similarity between the paired items. Wherever possible, this change was made in a central position to ensure that letters at morphemic boundaries remained the same. Pseudomorphemic and control nonwords were matched on number of letters, syllables, and orthographic neighbors, and summed log bigram frequency (see Table 1).[Table-anchor tbl1]

##### Procedure

The visual lexical-decision task was completed individually in a quiet room in school or at the university. Participants were instructed that they would be shown a series of words on the screen, and to indicate using a key press whether or not each was a real word that they knew, as quickly as possible. Participants were shown 12 practice items followed by the experimental items. Each trial began with a black fixation cross, which appeared in center of the screen for 1000ms, followed by the target, which appeared in lowercase Calibri font in the center of the screen until a response was made. For the practice items only, participants were given feedback on RTs and accuracy. Participants were given a short break after every 20 trials. The E-prime 2.0 program (Schneider, Eschman, & Zuccolotto, 2012a, 2012b) was used to present instructions and stimuli, and to record responses.

## Results

Table 2 summarizes performance by age group on background measures. Mean scores indicate performance that is close to test norms. Responses (accuracy and RTs) to nonwords in the visual lexical-decision task were analyzed. Inverse transformations were carried out on RTs to correct for distribution skews and transformed data were used throughout the analyses. RTs for incorrect responses were excluded, amounting to 25%, 23%, 15% and 12% of the data for children, younger adolescents, older adolescents and adults respectively. For the analysis, outliers were removed by excluding RTs that exceeded three standard deviations from the mean for that participant. Tables 3 and 4 show mean accuracy and mean RTs respectively for each nonword type by age group.[Table-anchor tbl2][Table-anchor tbl3][Table-anchor tbl4]

We used R (Version 3.3.0; R Development Core Team, 2016) and the lme4 package (Version 1.1–12; Bates, Maechler, Bolker, & Walker, 2016) to perform a generalized linear mixed-effects analysis of the effect of condition (pseudomorphemic vs. control) and age group (children vs. younger adolescents vs. older adolescents vs. adults) on the log odds of accuracy, and a linear mixed-effects analysis of the effect of condition and age group on RTs. For each analysis, condition, age group, and the interaction between condition and age group were entered into the model as fixed effects.^1^ We took a design-driven approach to determine the structure of random effects, starting with random intercepts by-participant and by-item, along with by-participant random slopes for the effect of condition and by-item random slopes for the effect of age group. Where a model failed to converge, or inspection of the correlations between intercepts and slopes of random effects indicated that the model was overparameterized, we simplified the random effects following recommendations from Baayen, Davidson, and Bates (2008). In each analysis, we analyzed 9,240 observations from 154 participants responding to 60 nonwords.

### Accuracy

The final model used for the analysis of accuracy was structured as follows: Model ← glmm (log.odds.accuracy ∼ Condition × Age group + (1|Participant) + (1|Item). Table 5 presents the output from this model.[Table-anchor tbl5]

The intercept represents the performance of the youngest age group (children) in the control condition; all other estimates are relative to this value. To determine whether the main effects of condition, age group and the condition x age group interaction were significant, pairwise likelihood ratio tests (LRTs) were used to compare the full model with simplified models in which the main effects were removed in turn. These comparisons indicated a significant effect of age group (LRT: χ^2^[6] = 61.44, *p* < .001), condition (LRT: χ^2^[4] = 47.48, *p* < .001) and a significant Age Group × Condition interaction (LRT: χ^2^[3] = 32.43, *p* < .001). The interaction between condition and age group was explored using the package phia (De Rosario-Martínez, 2015). An examination of simple effects revealed that the effect of condition was significant for children (χ^2^[1] = 6.81, *p* < .01), younger adolescents (χ^2^[1] = 11.04, *p* < .01), older adolescents (χ^2^[1] = 33.32, *p* < .001) and adults (χ^2^[1] = 23.90, *p* < .001). Examination of interaction contrasts showed that the magnitude of the effect of condition did not differ significantly between children and younger adolescents (χ^2^[1] = 1.70, *p* = .38), or between older adolescents and adults (χ^2^[1] = 0.51, *p* = .47), but the magnitude of the effect was significantly greater for older adolescents than for younger adolescents (χ^2^[1] = 13.48, *p* < .01).

### RTs

The final model used for the analysis of RTs was structured as follows: Model ← lmer (reaction time (RT).outliers.removed ∼ Condition × Age group + (1|Participant) + (1|Item). Table 6 presents the output from this model.[Table-anchor tbl6]

The intercept again represents the performance of the youngest age group (children) in the control condition and all other estimates are relative to this value. As before, we used pairwise LRTs to analyze the main effects of condition, age group and the condition x age group interaction. These comparisons indicated a significant effect of condition (LRT: χ^2^[4] = 70.65, *p* < .001), age group (LRT: χ^2^[6] = 164.00, *p* < .001), and a significant Age Group × Condition interaction (LRT: χ^2^[3] = 65.59, *p* < .001). The interaction between condition and age group was explored using the package phia (De Rosario-Martínez, 2015). An examination of simple effects revealed that the effect of condition was significant for older adolescents (χ^2^[1] = 12.37, *p* < .01) and adults (χ^2^[1] = 29.38, *p* < .001), but not for children (χ^2^[1] = 0.15, *p* = 1.00) or younger adolescents (χ^2^[1] = 0.10, *p* = 1.00). Examination of interaction contrasts showed that the magnitude of the effect of condition did not differ significantly between children and younger adolescents (χ^2^[1] = 0.78, *p* = .38), but the effect was greater for older adolescents than for younger adolescents (χ^2^[1] = 15.31, *p* < .001), and greater for adults than older adolescents (χ^2^[1] = 5.84, *p* < .05).

## Discussion

This study used a lexical-decision task to investigate the developmental trajectory of online morphological processing in nonword reading. Accuracy was lower for pseudomorphemic nonwords compared with control nonwords across all age groups; participants were more likely to incorrectly accept nonwords comprising a real stem and suffix (*earist*) than nonwords comprising a real stem and nonmorphological ending (*earilt*). This effect was greater in adults and older adolescents than in children and younger adolescents. The discrepancy in accuracy is consistent with existing adult findings (Crepaldi et al., 2010; Taft & Forster, 1975) and provides verification of morphological sensitivity in English-speaking children aged 7 to 9 (Burani et al., 2002; Casalis et al., 2015). The current study rectifies limitations in stimuli previously used with children (e.g., Burani et al., 2002; Casalis et al., 2015), and for the first time incorporates data from adolescent participants. Our findings are inconsistent with supralexical theories that see morphological analysis as taking place after lexical access (Giraudo & Grainger, 2001). Nonwords by definition are not represented in the lexicon. Therefore, if morphological structure is analyzed following lexical access, then there should be no difference in responses to pseudomorphemic (*earist*) and control nonwords (*earilt*) because both nonword types will be treated equally. Instead, our data lend support to morpho-orthographic theories that argue that the process of decomposition takes place prior to lexical access (Rastle & Davis, 2008; Taft, 2004), and dual-route models in which both whole-word access and decomposition are available (Baayen et al., 1997).

The RT data were less clear-cut. Both adults and older adolescents were slower to reject the pseudomorphemic nonwords (*earist*) relative to the control nonwords (*earilt*), replicating previous findings with adults (e.g., Crepaldi et al., 2010). This is consistent with Taft and Forster’s (1975) theory that complex words are stored in their root form in the lexicon, and are stripped of their affixes during recognition. A nonword comprising an existing stem and suffix (*earist*) will result in a lexical entry being retrieved (*ear*). The process of checking the legitimacy of the stem-suffix combination will generate longer RTs compared to nonmorphological nonwords (*earilt*), which are not decomposed and can be rejected once a search of the lexicon reveals no match. However, no difference in RTs was found for children and younger adolescents, corroborating findings from Casalis et al. (2015), which indicated that although French children were slower and less accurate to reject nonwords comprising a stem and suffix, the effect for English-speaking children was limited to accuracy.

Why might morphological effects emerge in accuracy but not RTs in children and younger adolescents? One possibility is that the types of suffixes used in the pseudomorphemic condition influenced response times. Previous studies with children have tended to include only neutral suffixes such as –*y* and –*er* (e.g., Carlisle & Stone, 2005; Laxon et al., 1992), which attach to independent words, do not alter stress in the word to which they attach, and are more productive than nonneutral suffixes such as –*ic* and –*ary* (Tyler & Nagy, 1989). The pseudomorphemic nonwords in the present study contained both neutral and nonneutral suffixes (60% and 40% respectively). It has been argued that the process of decomposition may vary according to suffix type (Hay, 2003) and there is some indication that children’s knowledge of these two types of suffix develops differently as they undergo a period of overgeneralization in the acquisition of neutral, but not nonneutral, suffixes (Tyler & Nagy, 1989). Thus, it is plausible that for the younger age groups, the morpheme interference effect on RTs only emerged for the more predictable, rule-driven neutrally suffixed pseudowords. However, subsequent analyses did not show this to be the case: the difference in RTs did not vary between the neutrally- and nonneutrally suffixed stimuli in either age group (all *p*s > .05).

A second possibility is that the mechanisms driving decomposition may differ between the younger and older age groups, and that children and younger adolescents might rely more heavily on explicit morphological knowledge in their decisions than the older participants. One argument raised by an anonymous reviewer is that the younger age groups may be more sensitive than the older age groups to the presence of an existing stem across both nonword types, independent of the morphological status of the nonword (see Casalis et al., 2015; Giraudo & Voga, 2016). This would slow responses to the control nonwords as well as the pseudomorphemic nonwords, which might account for the absence of an RT effect in the younger age groups. This would not explain the observed differences in accuracy, but slower responses to all nonwords could result in greater reliance on explicit processes to determine lexical status, leading to more errors in the pseudomorphemic condition.

Following the suggestion of a reviewer, we investigated the role of semantic interpretability to explore the idea that the younger age groups were relying more on explicit morphological knowledge than the older age groups. Semantic interpretability refers to the ease with which morphologically structured nonwords can be interpreted on the basis of the meanings of their morphological components (Longtin & Meunier, 2005). Nonwords such as *trueness* are semantically interpretable: the suffix –*ness* attaches to adjectives to form a noun, the stem-suffix combination is in accordance with English phonotactic rules, and there are equivalent real word examples (e.g., *gentleness*). All 30 pseudomorphemic nonwords were coded as either semantically interpretable or uninterpretable on the basis of the above criteria, resulting in 15 interpretable and 15 uninterpretable nonwords. We hypothesized that if children and younger adolescents were using explicit morphological knowledge, then they would make more errors rejecting semantically interpretable nonwords compared to uninterpretable nonwords relative to adults and older adolescents. However, post hoc analysis revealed that accuracy was lower for interpretable nonwords relative to uninterpretable nonwords across all age groups (all *p*s < .01), and further, that all age groups except the younger adolescents were slower to reject the interpretable nonwords relative to the uninterpretable nonwords (all *p*s ≤ .05).

On the surface, the influence of semantics may seem to lend support to supralexical theories of morphological decomposition, in which morphemic units are only accessed once whole-word lexical access has occurred. However, we would argue that the influence of semantic interpretability is reliant on the prior decomposition of morphologically structured nonwords: it is only through the separation of stem and suffix that the interpretability of the combination can be evaluated. Thus, it seems more plausible that the influence of semantics occurs following the process of decomposition. One limitation of the current study is that our measure does not allow a more direct exploration of this question. Lexical decision tasks do not make it possible to isolate processes relating to form-based decomposition and processes relating to meaning-based decomposition. Further, masked priming and ERP studies indicate that semantics do play a role in the later stages of word recognition (Lavric, Elchlepp, & Rastle, 2012; Rastle, Davis, Marslen-Wilson, & Tyler, 2000), and it is likely that the time taken to respond in a lexical-decision task will be sufficient for a semantic influence to emerge. In order to pinpoint the mechanisms driving morphological decomposition across development, future studies could adopt a masked priming approach to examine the time course of form- and meaning-based processing more closely.

It is clear from our findings that over the course of adolescence, there is some transition in how morphologically structured letter strings are processed during visual word recognition. This may reflect ongoing development and consolidation of tacit morphological knowledge, driven by increasing exposure to morphologically complex words across different contexts (Nagy et al., 2014). Specifically, adolescents encounter many morphologically complex words in academic texts that are not explicitly taught (Nagy & Anderson, 1984); therefore, the process of morphological decomposition may help to support comprehension. Further, according to Ehri’s (2005) stages of reading development, ‘chunking’ of grapheme-phoneme correspondences into larger units such as morphemes speeds sight word recognition. If chunking of suffixal units is slower to develop than chunking of lexical units, then this would support the idea that children and younger adolescents process the nonword stem initially, leading to slower RTs across both nonword types, whereas adults and older adolescents process morphologically structured nonwords as recognizable stem-suffix units. Thus, our findings may reflect an influence of automatized tacit morphological knowledge in the older age groups that has not yet emerged in the younger age groups.

It is likely that these changes are associated with the development of related skills, such as word reading and vocabulary. According to Nagy et al. (2014), sensitivity to morphemes in words should be linked to greater efficiency in reading those words. Meanwhile, vocabulary acquisition provides opportunities for exposure to the links between the orthography, phonology and semantics of morphemic units across different contexts (Reichle & Perfetti, 2003; Schreuder & Baayen, 1995). Although we did obtain measures of vocabulary and reading ability from our sample, we did not include these in our final models. In part, this was because they were not selected for the purpose of exploring these relationships. For example, our word reading efficiency measure comprised both monomorphemic and complex words, and our vocabulary measure captured depth of vocabulary knowledge rather than breadth (Ouellette, 2006). Arguably, vocabulary depth may not be as closely associated with tacit morphological knowledge as vocabulary breadth because it relates to the richness of semantic representations rather than multiple exposures to morphemic units across different contexts.

In conclusion, the older adolescent group responded to the nonword manipulation similarly to the skilled adult readers, indicating that, like adults, they rapidly process morphological structure. The younger adolescent group showed a similar pattern of results to the children: the accuracy data suggested some sensitivity to morphemic units, but there was little evidence that nonwords were processed at speed on the basis of morphological structure, as this effect did not emerge in RTs. Taken together, these results indicate some changes over the course of adolescence in the way morphologically structured letter strings are processed, which parallel continuing development in explicit morphological knowledge (e.g., Nippold & Sun, 2008), increasing exposure to morphologically complex words in different contexts (Nagy & Anderson, 1984), and ongoing changes in the cortex relating to visual word processing (Ben-Shachar, Dougherty, Deutsch, & Wandell, 2011). Further longitudinal investigation is warranted to track these transitions across the adolescent years and pinpoint the emergence of adult-like word recognition.

## Figures and Tables

**Table 1 tbl1:** Medians and Interquartile Ranges for Lexical Characteristics of Nonword Stimuli by Condition

	Pseudomorphemic		Control	
Lexical characteristics	*Mdn*	Interquartile range^a^	*Mdn*	Interquartile range
Number letters	7.00	1.75	7.00	1.75
Number syllables	2.00	1.00	2.00	1.00
Number orthographic neighbors	.00	.00	.00	.00
Summed log bigram frequency	15.98	4.17	15.10	4.65
^a^ Q3 – Q1.				

**Table 2 tbl2:** Means and Standard Deviations for Background Measures by Age Group

	Children		Younger adolescents		Older adolescents		Adults	
Measure	*M*	*SD*	*M*	*SD*	*M*	*SD*	*M*	*SD*
Nonverbal ability^a^	48.22	9.35	49.51	8.40	50.26	7.36	48.13	11.06
Oral vocabulary^a^	51.88	7.82	52.92	8.67	55.03	7.39	56.90	6.45
Sight word efficiency^b^	106.34	9.98	101.35	14.47	101.94	9.78	109.65	12.82
Phonemic decoding efficiency^b^	103.94	10.81	103.24	14.16	104.35	10.60	108.74	8.75
^a^ *T* scores: *M* = 50; *SD* = 10. ^b^ Standard scores: *M* = 100; *SD* = 15.								

**Table 3 tbl3:** Raw Means and Standard Errors for Percentage Accuracy by Condition and Age Group

	Condition			
	Pseudomorphemic		Control	
Age group	*M*	*SE*	*M*	*SE*
Children	69.87	2.10	80.00	2.13
Younger adolescents	71.35	2.43	83.60	2.17
Older adolescents	76.94	1.85	93.15	1.16
Adults	81.29	2.26	93.87	1.28

**Table 4 tbl4:** Raw Means and Standard Errors for Reaction Times by Condition and Age Group

	Condition			
	Pseudomorphemic		Control	
Age group	*M*	*SE*	*M*	*SE*
Children	1925.05	100.14	2002.75	114.33
Younger adolescents	1130.62	64.59	1134.40	64.47
Older adolescents	859.06	34.89	786.94	23.92
Adults	743.25	26.63	678.43	22.76
*Note*. Reaction times for correct responses only (untransformed and untrimmed).				

**Table 5 tbl5:** Output for Accuracy Model

Fixed effects	Estimate	*SE*	*z*
Intercept	1.69	.20	8.31***
Pseudomorphemic condition	−.60	.23	−2.61**
Younger adolescents	.29	.22	1.33
Older adolescents	1.31	.23	5.59***
Adults	1.53	.25	6.07***
Pseudomorphemic condition: Younger adolescents	−.19	.15	−1.30
Pseudomorphemic condition: Older adolescents	−.88	.18	−5.03***
Pseudomorphemic condition: Adults	−.72	.19	−3.70***
** *p* < .01. *** *p* < .001.			

**Table 6 tbl6:** Output for RT Model

Fixed effects	Estimate	*SE*	*t*^a^
Intercept	.69	.04	16.90***
Pseudomorphemic condition	.01	.02	.39
Younger adolescents	.36	.06	6.08***
Older adolescents	.69	.06	11.72***
Adults	.89	.06	14.33***
Pseudomorphemic condition: Younger adolescents	−.02	.02	−.88
Pseudomorphemic condition: Older adolescents	−.10	.02	−5.06***
Pseudomorphemic condition: Adults	−.14	.02	−7.43***
*Note*. RT = reaction time.			
^a^ Degrees of freedom and *p* values were calculated using Satterthwaite approximations.			
*** *p* < .001.			

## Footnotes

1 Incorporating performance on background measures of reading and vocabulary in models examining accuracy resulted in a failure to converge, indicating that our data lacked sufficient power to explore individual differences. Thus, our final models included just the fixed effects of condition, age and their interaction.

## Nonword Stimuli

**Table:**

Pseudomorphemic	Control
antism	antilm
bandary	bandady
beanish	beanith
begence	begenge
boltous	boltoes
classous	classoes
coldity	coldidy
earist	earilt
elbowism	elbowilm
flipory	flipody
freeness	freenels
gasful	gasfil
gumful	gumfil
habitic	habitig
happenance	happenange
illist	illilt
jawly	jawla
lidary	lidady
meltance	meltange
mouthize	mouthime
opposement	opposemant
passment	passmant
poority	pooridy
ripence	ripenge
sheeter	sheetel
socketer	socketel
towerly	towerla
treasonize	treasonime
trueness	truenels
wigish	wigith
